# Transmasculine individuals’ experiences with lactation, chestfeeding, and gender identity: a qualitative study

**DOI:** 10.1186/s12884-016-0907-y

**Published:** 2016-05-16

**Authors:** Trevor MacDonald, Joy Noel-Weiss, Diana West, Michelle Walks, MaryLynne Biener, Alanna Kibbe, Elizabeth Myler

**Affiliations:** Community advocate, Winnipeg, MB Canada; School of Nursing, University of Ottawa, Ottawa, ON Canada; Diana West Lactation Services, Long Valley, NJ USA; Community, Culture, & Global Studies, University of British Columbia, Kelowna, BC Canada; The Newman Breastfeeding Clinic, Toronto, ON Canada; Seventh Generation Midwives Toronto, Sunnybrook Health Sciences Centre, Toronto, ON Canada; Mahala Breastfeeding Center, Hackettstown, NJ USA

**Keywords:** Breastfeeding, Chestfeeding, Chest masculinization surgery, FtM, Gender dysphoria, Lactation, Top surgery, Trans, Transgender, Transmasculine

## Abstract

**Background:**

Transmasculine individuals are people who were assigned as female at birth, but identify on the male side of the gender spectrum. They might choose to use and engage their bodies to be pregnant, birth a baby, and chestfeed. This study asked an open research question, “What are the experiences of transmasculine individuals with pregnancy, birthing, and feeding their newborns?”

**Methods:**

Participants who self-identified as transmasculine and had experienced or were experiencing pregnancy, birth, and infant feeding were recruited through the internet and interviewed. Interviews were transcribed verbatim. We used interpretive description methodology to analyze the data. Our analysis was guided by our awareness of concepts and history important to the transgender community.

**Results:**

Out of 22 participants, 16 chose to chestfeed for some period of time, four participants did not attempt chestfeeding, and two had not reached the point of infant feeding (i.e., were still pregnant or had a miscarriage). Nine of the 22 study participants had chest masculinization surgery before conceiving their babies. Six participants had the surgery after their children were born, five desired the surgery in the future, and two did not want it at all.

Chest care, lactation, and chestfeeding in the context of being a transgender person are reported in this paper. The participants’ experiences of gender dysphoria, chest masculinization surgery before pregnancy or after weaning, accessing lactation care as a transmasculine person, and the question of restarting testosterone emerged as data. We present the participants’ experiences in a chronological pattern with the categories of before pregnancy, pregnancy, postpartum (6 weeks post birth), and later stage (beyond 6 weeks).

**Conclusions:**

The majority of participants chose to chestfeed while some did not due to physical or mental health reasons. Care providers should communicate an understanding of gender dysphoria and transgender identities in order to build patient trust and provide competent care. Further, health care providers need to be knowledgeable about lactation and chest care following chest masculinization surgery and during binding, regardless of the chosen feeding method and through all stages: before pregnancy, during pregnancy, postpartum, and afterward.

**Electronic supplementary material:**

The online version of this article (doi:10.1186/s12884-016-0907-y) contains supplementary material, which is available to authorized users.

## Background

### Introduction and purpose

Transmasculine individuals are people who were assigned as female at birth, but identify on the male side of the gender spectrum. Their anatomical birth sex does not match their inner sense of gender identity. This incongruity may be a source of gender dysphoria, the experience of distress or anxiety regarding one’s gender and body. To transition is to make a change in one’s public gender identity, whereas one’s inner gender identity may have been the same since birth. Transitioning may encompass changes in name and clothing choices; using testosterone; or having surgical procedures related to the chest or reproductive organs. The gender binary is the assumption that there are two distinct and opposite genders, rather than a gender spectrum [1, 2].

Taking testosterone results in changes to secondary sex characteristics such as increased muscle mass, fat re-distribution, growth of facial hair, male-pattern baldness, and deepening of the voice, and it usually causes the cessation of menses [3–5], though some transmasculine individuals may become pregnant while taking testosterone [5]. For some individuals, some of these body changes are permanent [3, 4]. Cessation of testosterone therapy can result in the return of menses and fertility [3].

Some transmasculine individuals choose to engage their bodies to carry a pregnancy and birth a baby. They also make choices about how they will feed their newborns. Whether they have taken steps to transition medically or surgically and whether or not they choose to chestfeed their babies, these individuals may require assistance from lactation professionals.

We asked the question, “What are the experiences of transmasculine individuals with pregnancy, birthing, and feeding their newborns?” We interviewed 22 individuals who self-identified as transmasculine and who had experienced pregnancy, birth, and infant feeding, or were currently experiencing pregnancy and planning for birth and infant feeding. Based on findings from the research study, the purposes of this paper are: 1) to describe transmasculine individuals’ experiences with their chests, lactation, and chestfeeding; 2) to inform transmasculine individuals who might want to chestfeed their babies; and 3) to guide health care professionals (e.g., lactation consultants, midwives, nurses, physicians, and surgeons) who provide breast and chest care. We report our research findings regarding pregnancy and birth in a separate paper.

### Inclusive language

Language, both written and spoken, has power. Words can heal or wound, uplift or disparage. During the interviews, participants expressed the importance of words related to gender. Being described with words such as *she, her, mom*, *mum*, *mother, breasts,* or *breastfeeding* could be distressing for a parent who self-identifies differently. A finding of this paper is that health care providers (HPCs) and others may inadvertently cause harm and intensify feelings of gender dysphoria by misgendering transmasculine patients. Conversely, care providers can affirm a patient’s gender identity by gendering them correctly, and thus alleviate distress related to gender dysphoria. To uphold the importance of using respectful and appropriate language, inclusive and gender-neutral terms are used in this work. In order to be inclusive of all gender identities, we use the pronouns *they*, *them*, and *their* to refer to an individual when appropriate, as opposed to limiting these pronouns to plural use.

Of the 22 participants, 20 participants said they preferred male pronouns and two preferred neutral pronouns or both “he” and “she.” All participants referred to their upper front torso as their *chest* and avoided the term *breasts*. However, two participants spoke of their breasts when they described what it was like to breastfeed their babies much earlier in their lives, before they had come out as transgender. In general, in this paper, the term *chest* is used to refer to the upper front torso, which includes the nipples and surrounding skin, mammary tissue, and fatty tissue.

Study participants used a range of terminology to discuss feeding their babies from their chests. Six used the term *breastfeeding*, four spoke of *nursing*, three preferred to say *chestfeeding*, two used both *breastfeeding* and *nursing*, two said it did not matter, one used *feeding* and *nursing*, and one used *feeding* and *mammal feeding*. In this paper, the term *chestfeeding* is used to refer to transmasculine or gender non-conforming individuals and the act of feeding a baby or child at the chest with or without a supplementing tube. We use *breastfeeding* with regard to research or settings that are not inclusive of transgender or gender non-conforming individuals.

Breastfeeding is not exclusive to cisgender women (i.e., women whose gender identity and gender expression conform to the gender that is typically associated with their birth-assigned sex) who gave birth and are lactating. Transmasculine individuals also lactate and chestfeed babies they have birthed. Individuals sometimes induce lactation or relactate to feed babies and children they did not birth. In some cases, a supplementing tube provides nourishment while breastfeeding or chestfeeding. A supplementer could be used by women, transmasculine individuals, or gender non-conforming persons who have given birth, are lactating, and need to supplement, or by individuals who chose to relactate or induce lactation to breastfeed or chestfeed a baby or child.

### Literature review

When it comes to building knowledge about health care, transgender issues are often folded into discussions about gay, lesbian, and bisexual health rather than treated separately [6–8]. The research literature from the past 10 years specific to transgender health consists primarily of general health needs assessments and research studies [9–11]. However, physicians and nurses have begun to contribute with wide-ranging topics such as identifying physician-side barriers to providing healthcare for transgender patients; curriculum recommendations for health care students; and analyses about gendered language, the invisibility of transgender diversity, and transgender patients’ avoidance of emergency care [12–17].

There are a few journal articles about transgender men and gynecological care [18, 19]. Dutton and Fennie interviewed six transgender men to determine their gynecological needs [19]. In their qualitative study, breasts were identified as causing significant discomfort relating to gender dysphoria [19]. Dutton and Fennie recommended that clinicians should encourage clients to asexualize the breasts [19], although it is unclear how this strategy might be implemented.

There are commentaries and research about transgender individuals’ experiences of pregnancy and birth [20–22], yet we found little in the academic literature specifically about transmasculine individuals and chestfeeding, with the exception of two papers published in 2015. Farrow wrote a commentary about lactation support and the lesbian, gay, bisexual, transgender, queer, questioning, and intersex community that included transgender men and infant feeding [23]. Wolfe-Roubatis and Spatz presented three cases and summarized strategies to provide support for transgender men who are lactating [24]. While commentaries and practical suggestions bring the topic to the attention of health care providers, there is a lack of research about transmasculine individuals’ experiences with chestfeeding. With this paper, we aim to begin to establish a body of knowledge about transmasculine individuals and their experiences of lactation and chestfeeding.

## Methods

### Study design

Because of the current lack of research on this topic, we chose a broad research question: “What are the experiences of transmasculine individuals with pregnancy, birth, and infant feeding?” We used a qualitative methodology to design this study and interpretive description methodology to analyze the data. According to Thorne et al., “interpretive description acknowledges the constructed and contextual nature of human experience that at the same time allows for shared realities” [25] (p. 172). We wanted to learn about the study participants’ choices and experiences with infant feeding. The research was designed as semi-structured interviews with a goal of describing and interpreting patterns and themes that emerged from the data.

### Recruitment

Inclusion criteria for the main study specified individuals who self-identified as transmasculine regardless of stage of transition. Participants were required to have experienced or to be currently experiencing pregnancy and birth, and there was no requirement regarding type of infant feeding. Inclusion was not restricted by geography, but all interviews were conducted in English.

We recruited a convenience sample of participants mostly through the internet. A letter and a poster were used to recruit participants. The letter was posted to the Facebook page, “Birthing and Breast or Chestfeeding Trans People and Allies,” (formerly “Birthing and Breastfeeding Transmen and Allies”) managed by the first author (TMD). The poster was circulated through email.

### Data collection

TMD used a questionnaire to collect demographic and descriptive information about participants before each interview. He then used a semi-structured interview guide that included a list of open-ended questions and prompts. The questionnaire used to collect demographics information and the semi-structured question guide used as a basis for interviews are included in Additional file 1. Interviews were conducted by Skype or telephone. To allow for personal preference and to ensure convenience, safety, privacy, and confidentiality, each participant chose the time of their interview and whether it would be a telephone call or a Skype call with cameras. All interviews were audiotaped using a digital recorder, and then transcribed verbatim, de-identified, and shared with the team to consider themes that emerged as the interviews were conducted. Interviews continued until it was determined we had a wide variety of experiences with consistent, common topics.

### Data analysis

The transcribed interviews were uploaded to NVivo 10 and JN-W, MW, and TMD coded data. We used a combination of line-by-line coding and overall analyses to identify associations, patterns, and relationships. These steps were guided by qualitative description analyses methods [26–28]. All interviews were coded by at least two researchers. Each used their own topics and then topics were merged. The topics that emerged from the data about infant feeding were well-defined and practical. After the topics had been identified from the interviews, we reversed the process and the sets of approved quotes were recoded under the topics to ensure we had not missed any key topics.

Methodologically, we based our analysis on interpretive description analysis. Thorne, Reimer Kirkham, and O’Flynn-Magee state, “Interpretive description departs from traditional qualitative descriptive approaches in that it assumes nurse investigators are rarely satisfied with description alone and are always exploring meanings and explanations that may yield application implications” [29] (p. 6). We wanted an analysis that would provide a rich description while at the same time giving us an interpretation that would lead to recommendations for transmasculine individuals considering pregnancy or chestfeeding and for HCPs who work with transgender patients and clients.

## Results

### Description of the participants

We had a sample of 22 participants ranging in age from 24 to 50 years. Nineteen spoke English as a first language, and three cited both English and French or another language as their first languages. Fifteen were living with a partner at the time of their interview, and 17 participants had a family income of over $40,000 CAD per year. Every study participant had at least some college-level education, and 13 participants had graduate degrees. Participants were located in North America, Europe, and Australia.

For ethnicity, we used an open question and invited participants to use their own descriptive words. Thirty-nine different words were used by the 22 participants to describe their ethnicity. Among the 22 participants, 11 used a single word, six used two words, four used three words, and one participant used four words to describe ethnicity. Thirteen participants used race as a means of identifying ethnicity: ten White or Caucasian; two Black; and one non-Hispanic. Nationality was cited 22 times with five participants combining two or more terms to describe themselves: Aboriginal, Albanian, American (2), British, Canadian (2), Cajun, Cree, Creole, Czech, English (2), European (2), French, German, Greek, Irish, Italian, Ukrainian, and Welsh. Three used Jewish and one used a country, Australia, to describe their background.

We did not ask specifically about disabilities in our demographics questionnaire, but during interviews, two participants mentioned that they were disabled or had a partner or close friend with a disability who was involved in their pregnancy and birth.

Twenty study participants had carried babies to term at the time of their interviews. Fourteen participants had one child. Eight participants reported on the initial questionnaire that they had experienced miscarriage and during interviews two participants mentioned that they had an abortion. Two participants mentioned during interviews that their pregnancies were not planned. Pregnancy lengths and birth weights were unremarkable: births occurred between 36 and 42 weeks gestation and birth weights ranged from 2722 to 4308 g. Seven participants reported experiencing postpartum depression, of whom three participants received a medical diagnosis. Thirteen participants reported no symptoms of postpartum depression and two had not reached the postpartum stage. Nine participants had taken testosterone before they conceived, eight had started taking it for the first time after their children were born, and five had never taken it.

Sixteen study participants chose to chestfeed for some period of time, four participants did not attempt chestfeeding, and two had not reached the point of infant feeding (i.e., they were still pregnant or had a miscarriage). Nine of the 22 study participants had chest masculinization surgery before conceiving their babies. Six participants had chest masculinization surgery after their children were born, five desired the surgery in the future, and two did not want it at all.

### Participants’ experiences

We present the participants’ experiences in a chronological pattern with the categories of before pregnancy, prenatal, postpartum (6 weeks post birth), and later stage (after 6 weeks postpartum). This format follows the narrative flow of the interviews and provides a rich description of the participants’ stories.

#### Before pregnancy

Participants cited chest-related gender dysphoria as driving an urgent need for chest masculinization surgery. In a certain context, gender dysphoria refers to a medical diagnosis from the DSM-5 of an individual who displays “a marked incongruence between one’s experienced/expressed gender and assigned gender” [30] 302.85 (F64.1) and experiences discomfort as a result. In this paper, we use the term gender dysphoria to denote transgender individuals’ lived experiences of distress about their bodies as they describe these experiences.

Chest masculinization surgery, also known as top surgery or male chest-contouring surgery, refers to a procedure to remove mammary tissue and create a masculine chest. It is not the same as a breast reduction where the goal is to produce smaller but still recognizably female-shaped breasts. It also differs from a mastectomy performed to prevent or treat cancer. Chest masculinization surgery does not normally involve removal of all mammary tissue since doing so would result in a sunken-looking chest.

Whether or not a participant had chest masculinization surgery before conceiving and the type of chest masculinization surgery were main factors that affected decisions and experiences around lactation and feeding. Out of the nine study participants who had chest masculinization surgery prior to their first pregnancies, five participants reported that they knew at the time of surgery that they eventually wanted to have children; four participants said they had not considered pregnancy or lactation at the time of surgery.

Although surgical techniques and their names varied, the techniques as they were reported by participants could be grouped into two categories: a) surgeries that involved two broad incisions underneath the mammary tissue and temporary removal and subsequent repositioning of the nipples using nipple grafts (often called “double incision with nipple grafts”) and; b) surgeries that removed mammary tissue while preserving the nipple pedicles. Five participants had the “double incision” style surgery with nipple grafts. Three participants had surgeries that preserved the nipple pedicles: one had a “keyhole” or “purse-string” type procedure where incisions bordered the areolae and the nipples were not moved; one had a double incision style surgery but the nipples were not re-positioned; and one had a procedure similar to the double incision but a pedicle flap was used to avoid the need for nipple grafts and to maintain intact nipple stalks. One participant did not discuss the type of surgery he had.

Participants were asked about their decisions regarding chest masculinization surgery. Reasons for having the surgery included chest-related gender dysphoria and being identified as female due to having a female-appearing chest. Participants also cited ongoing discomfort from binding, a term used to describe the practice of flattening the chest with material such as bandage wrappings or tight, stretchy garments. In all cases, chest masculinization surgery seemed to provide immense relief from these experiences. Two participants mentioned that the dramatic lessening of gender dysphoria brought about by chest masculinization surgery facilitated the choice to become pregnant, and these participants noted that the level of gender dysphoria during pregnancy would have been unbearable if they had female-appearing mammary tissue.

I should know [the type of chest masculinization surgery] and I don’t. That tells you how much I was paying attention. I was like just get them off me, I don’t care what you do. Just get them off me. [Cory, who initially could not recall the surgical technique used for his chest masculinization surgery but later in the interview determined on the basis of his scars that it must have been the double incision]

It [chest masculinization surgery] was the thing that made it possible for me to get pregnant… and I’d never been so depressed in that time that I tried to get pregnant without top surgery… I literally had nightmares of cutting my chest off with scissors… I assumed that I couldn’t [chestfeed]. I mean it was hard because it was one of the first things my mom had said to me after I told her about the surgery—“Well, you’re never going to be able to feed your babies and that’s terrible.” And I was like, “Maybe I don’t wanna feed my babies that way.” It was a point of defensiveness with me that I could still have a relationship with my child without feeding them from my chest. [Felix, double incision surgery]

I don’t think that I could have navigated this entire process [pregnancy] without having had top surgery. [Oren]

Participants did not discuss future infant feeding choices with their surgeons in consultations prior to surgery, and no surgeon brought up the topic. Participants believed that their surgeons subscribed to a binary view of gender, and that pregnancy and chestfeeding would not fit with their surgeons’ ideas of what a “true” transgender man would want to do. To reveal that they were considering future pregnancies or chestfeeding might delay or jeopardize their chance to have chest masculinization surgery.

I didn’t want to wait. I was on a waiting list of where to get it covered and I didn’t want to wait anymore ’cause I’d been binding for 6 years…I went to this plastic surgeon dude and he hadn’t done a lot of trans surgeries but we paid for it… He was just like, “Oh yeah, you’re the guy trapped in a girl’s body, and now your body matches how you feel inside.” And I was like, “Uh, yeah, sure, thanks, I’ll take my new chest now and go.” [Kai]

The “born in the wrong body” or “trapped in the wrong body” narrative is a simplistic depiction of transgender people that is commonly found in media stories [31]. Proponents of this narrative give the impression that transgender people want to change all aspects of themselves to conform absolutely to the opposite traditional gender role and physical sex from what they were assigned at birth, an assumption that would logically exclude transmasculine bodies from the realm of pregnancy and lactation.

Four study participants specifically mentioned in interviews that they felt unable to discuss future pregnancy or lactation with their surgeon before chest masculinization surgery due to the surgeon’s belief in the gender binary or the “trapped in the wrong body” narrative. One of these participants explained that he postponed asking necessary health-related questions until after surgery because he was unsure of his surgeon’s views about gender identity.

To get top surgery you kind of have to talk about it as if you never want to get pregnant, right, to the surgeon. You can’t be wiggly about that so I don’t know… I don’t think there was a way to talk to him about getting surgery without having a conventional narrative about it. [Felix]

After surgery, because again I didn’t know what his [the surgeon’s] thought processes were about gender identity, afterwards I called him up and I asked him. I wasn’t really thinking about nursing, but more the worry of are they going to grow back if I get pregnant. [Oren]

Four participants were certain they would not be able to chestfeed for physical reasons because they had chosen chest masculinization surgery. They cited physical pain, inability to make milk, and lack of tissue to latch to as a result of surgery as factors that would make chestfeeding impossible.

I had top surgery a few years ago, so I chose then that if I ever wanted to have a child I wouldn’t be able to breastfeed. So I have a masculine chest and a formula, bottle-feeding baby…my choice around top surgery was one around which I felt incredibly disconnected to my chest, so much so that I literally had no sensation at all. [Dagan]

I think the biggest issues [with chestfeeding a baby after chest masculinization surgery] may be physical as opposed to psychological. After surgery it took my chest about a year to get sensation back and it’s kind of hit or miss. There are times when somebody touches it and it kind of makes me nauseous…There’s this fine line between I can feel it and it hurts, so I don’t know. [Kennedy]

On the other hand, seven participants explained that they knew well before conception that they wanted to chestfeed their babies. They had family members who breastfed or they had learned about how human milk provides optimal nutrition and how chestfeeding is a way to build a secure attachment between baby and parent. Five participants mentioned that they delayed chest masculinization surgery in part or wholly because of their commitment to chestfeeding.

It [breastfeeding] is the absolute, well, first my mother did. Um, and with the information that was available at the time, it was the best thing to do for your baby. [Nick, came out and transitioned after weaning]

My partner used to go out with a midwife. Her girlfriend was learning to be a midwife while they were together…So, she learned a lot of stuff around chestfeeding. I was really surprised to learn all the things about, the kind of stuff like how your body can sense what is in the room and what your baby needs antibodies to and your body can bump it up and produce it in the milk. That to me, when I heard those kind of things, it was just incredible that your body could do that kind of thing. [Peter]

I always, I wanted it [chest masculinization surgery], since transitioning, but because of being able to feed my kids, I might have waited or I might not have [speculating because surgery was not financially possible]. [Vince]

#### Prenatal

During pregnancy, participants noticed and coped with chest changes and planned for infant feeding. A wide range of experiences were reported in terms of mammary tissue growth as well as related gender dysphoria. Six of the participants who had previous chest masculinization surgery found that their chest tissue was growing back some amount or even to pre-surgery size. Those who had not had surgery experienced predictable chest growth.

Out of nine participants who had previous chest masculinization surgery, two reported no change to their chest tissue during pregnancy. Both participants had the double incision surgery with nipple grafts. Three participants reported minor chest tissue changes: two had double incision surgery with nipple grafts and one had double incision surgery with a pedicle flap. One participant who had double incision surgery with nipple grafts reported some degree of chest tissue changes. Two participants stated they experienced significant chest changes during pregnancy and that their chest tissue had grown back to its pre-surgical size. One of these participants had liposuction and keyhole surgery and the other had double incision surgery without repositioning of the nipples.

I was under the complete understanding that it was permanent, that it was not ever going to grow back, so actually it was quite the opposite—the fact that it did grow back [during pregnancy] was really…I would have done a lot better if I had been warned that that was possible that that would happen. [Emmett, previous chest masculinization surgery, double incision with no nipple grafts or re-positioning]

The experience of gender dysphoria was a common theme during pregnancy due to body changes, but it was not experienced by all participants, and no two stories were the same. While participants identified many bodily sources of gender dysphoria including changing hormones, widening hips, and reduced facial hair, breast or chest-related gender dysphoria was cited frequently. Some participants described gender dysphoria as being simply about their own relationship with their body.

I kind of feel like the whole pregnancy and certainly birth and now feeding him it’s like I feel like I don’t really have a body. It’s not my own. I’m not sure if that’s a good or a bad thing but it just feels like I don’t really own this body currently. So I guess to kind of answer your question more, because it’s really hard, I don’t really feel like myself, when I look at myself I don’t look like myself and it was like prior to being pregnant and having him I did a certain amount of binding to my chest and that’s been really difficult to not do that anymore and I feel very floppy all over the place. [Julian]

Nine study participants stated that they did not experience an increase in gender dysphoria related to body changes during pregnancy, but they did experience distress when others misgendered them as a result of their pregnancies or body changes. Participants described situations where growth of chest tissue affected how their gender was perceived by others, which then triggered their own gender dysphoria. Having prominent chest tissue seemed to result in individuals being identified by others as female more often than other typically female secondary sex characteristics, including a pregnant belly. Participants noted that their pregnant bellies were frequently identified as fat, male bellies, and were generally easier to disguise with clothing than chests were.

My gender identity is still male [during pregnancy] but it’s hard really because I believe my gender is also part me and part society. So I think it’s, I’m both, and so if society is not seeing it then I don’t really consider myself there all the way, because I think it’s kind of a two-way street there…Identity is not something that is separate from my family or my community. [Emmett, previous chest masculinization surgery, experienced significant reversal of body changes during pregnancy including reduced facial hair, higher-pitched voice, and re-growth of chest]

I was pregnant, I was like seriously pregnant, and then they [nurse at clinic] called me but, of course, they call the female name [participant’s legal name was female at the time] and we get up and we walk over and the nurse hands the [specimen] cup to my female partner, who doesn’t look pregnant at all, she’s like very tall and thin, and then says “Oh, here, I need this sample, and your husband can wait over there,” and is like waving me off, this short, fat man, and it’s like wait, no, it’s my husband that’s actually pregnant. [Daniel]

Four participants who had not had chest masculinization surgery mentioned that they had practiced chest binding before they conceived their babies, but no participant reported being able to bind during pregnancy. Those who tried binding found it too painful or inadequate to continue, which also contributed to chest-related gender dysphoria. One participant who did have previous chest masculinization surgery attempted binding because his mammary tissue increased during pregnancy but he stated that the practice was ineffective for him.

So there was some swelling and I got binders again, which I had gotten rid of years before, and just folded them up so they would be over my belly, but in the end I stopped using it because it didn’t feel like—it wasn’t enough. [Felix, previous chest surgery, double incision with nipple grafts]

I stopped binding pretty early because it was also very tender. I didn’t go back [to binding] until I was finished feeding my kids…between pregnancies I went back to it but, but yeah, I mean that’s pretty much how I coped. I just tried to let go of my usual way of doing things. I think that was another step towards not passing as much and sort of, I don’t know, realizing that I was going to have to make some compromises from the way that I would prefer to do things because of the choice that I had made. [Vince, no chest masculinization surgery]

The temporary nature of pregnancy and chestfeeding came up often during interviews as a means of coping with chest-related gender dysphoria. Participants anticipated an eventual end to pregnancy and lactation, and planned for future chest masculinization surgery or surgical revisions. Revision surgery is commonly performed at a minimum of 6 months after chest masculinization surgery to ameliorate the appearance of scars, remove excess tissue, improve symmetry, and adjust nipple and areolar dimensions [32]. Utility and purpose were also important as transmasculine individuals reminded themselves that body parts normally associated only with distress would soon provide nutrition for their babies.

At the time I was just like it’s OK, now is not forever. I’ve always wanted a revision and I never got one so the way I got myself through it [pregnancy with mammary tissue growing back] is I was just like it’s OK because I was planning on getting the revision anyways. [Kai, previous liposuction and keyhole surgery]

I just really felt like I’d been stuck in this body, I’d hated it my whole life and here was just this one amazing thing that my chest could do that you know I sort of felt like that’s maybe the reason why I had been given this body. So I just felt like it was something that I wanted to do. [Peter, no previous chest masculinization surgery]

With the breasts, it was like what do I have these for anyway. The functional aspect of it to me, that made the most sense to me. I guess on some bizarre level I just wanted to feel like there was some reason I have this body. I guess having, being equipped with a body that was female, it seemed like that was a reasonable thing to do, was to use the breasts to feed the baby. [Daniel, no previous chest masculinization surgery]

Post-surgical participants assessed their chests for changes during their pregnancies to try to determine if chestfeeding would be realistic. They had concerns about milk production and the ability to latch a baby to a chest with little tissue.

My first indication that I was pregnant, even on the miscarriage, was that my breasts were tender, my nipples were tender. Or at least on my right side I was. My left side—both of my nipples were made from one nipple—and so the one on my right side is more I guess whole, or has more integrity than the left, so the one on the left I never had much happen but the one on the right I did. And then I actually had a little bit of swelling I think. There was no permanent change but I definitely had sensitivity so much so that I really thought I might be able to produce a little bit of milk. [Cory, previous double incision with nipple grafts]

I am pretty sure that, well, my chest tissue hasn’t grown back. It grew back right at the start of the pregnancy a small amount just here and then it stopped. I’m pretty sure that, I’ve done some reading, and I’m worried about the baby being able to latch on, there being enough tissue to latch on. [Travis, previous double incision chest masculinization surgery]

During their pregnancies, participants planned ahead for infant feeding and sought information and support for their choices. All study participants who had not had previous chest masculinization surgery chose to chestfeed their infants, and seven participants described it as a simple decision due to what they considered health benefits and the utility of chestfeeding. Four participants also mentioned bonding and attachment parenting as reasons to chestfeed their babies. Participants cited aspects of their identities and circumstances other than identifying as transmasculine when they described how they arrived at their decisions. They discussed racialized communities, parenting and breastfeeding groups, urban versus rural settings, and other family members’ infant feeding choices as contributing significantly in terms of their decision.

I always knew I would breastfeed. I had known that there are a lot of benefits to breastfeeding. It wasn’t even a choice, I just knew. I was fortunate that I could. [Alex, came out and transitioned after weaning his child]

We happen to live in a place where there are all these hippies and we shop at a co-op, and everyone is breastfeeding like crazy. There’s a store that sells cloth diapers up my street. We just happened to move into the most progressive area ever. When I meet people they recognize that I’m transgender without me even saying anything half the time…Here, you’re much more likely to hear about breastfeeding or at least see it…We talk about—in the black community, in cities—there can be this overwhelming feeling that it’s not socially acceptable. I’ve heard people say that, “We don’t do that because that is what slaves did.” [Adam, multiracial family]

One of the most supportive communities I found during my pregnancy was the La Leche League community, which my midwife was heavily involved in. And she got me connected to a lot of other people and they were just so accepting and warm and gracious…I also really wanted that relationship with my child. I wanted that connection, you know, of holding my child, my own child, to my chest, being connected in that way and being able to offer that not just nourishing but nurturing aspect. [Cory, previous double incision with nipple grafts]

I did read up a lot about nursing for example. I don’t know how it’s like now, I think North America in general has become a bit more breastfeeding friendly, but you know 15 years ago it was just starting to come back into the public landscape and there was still a lot of push against it. That’s where I did take charge because I wanted to inform myself because I knew I was going to face some resistance possibly from family members or health care providers. [Gabby, experienced pregnancy before coming out or transitioning]

Six participants mentioned that they experienced significant pressure from health care providers, friends, or family to chestfeed their babies. Two participants reported that their decisions about chestfeeding affected their legal custody rights as parents. When midwives and doulas were explicitly pro-chestfeeding and seemed obviously pleased that their clients did choose to chestfeed, participants described a greater sense of anxiety and pressure to succeed.

My lawyer very strongly suggested that I do it because it’s a very….If you’re breastfeeding the child they [social services] are less likely to take it away from you. So she [lawyer] very much pushed, “You have to breastfeed—you have to prove that you’re breastfeeding. If possible do it in front of the social workers when they come around.” I was just, like, I’d really rather not. I planned on pumping and then giving the child the breast milk but not actually full on doing it. But I had to. [Ben, no previous chest surgery]

Because I’m not a mom…and I wasn’t breastfeeding or chestfeeding, for me, there wasn’t any assumption that this kid needed to be with me. The idea was you could separate him from me earlier on [in order to share custody], but all the hormones from giving birth, all the other connections are still there, and it was incredibly painful. [Felix, double incision surgery]

People who know that I’ve had—I call it a double mastectomy to them because that’s the easiest—and they see my chest, they see everything and then they ask me about breastfeeding. Midwives and doulas, like that’s a big thing. And I’m like I don’t have any breasts. It’s not even that I have a little bit of tissue. I have nothing, it’s not there. The nipples were removed and put back. I really don’t think that that’s going to happen. [Oren]

I’ve sort of run into health workers who are trying to be respectful saying, “So how do you want to feed?” But then as soon as I say I’m going to nurse they’re like, “Oh, good,” which then puts me in a position that like if I ever want to change my mind I know they’re going to be disappointed in me or whatever it is, like it puts the pressure on. [Lee]

#### Postpartum

The postpartum period involved a variety of lactation and chest-related challenges and experiences. Seven of the 16 participants who initiated chestfeeding reported that they experienced gender dysphoria while chestfeeding. Five of these individuals found means of coping with gender dysphoria such as making use of layered clothing or thinking in terms of the utility of chestfeeding. For two of the participants, intense and overwhelming gender dysphoria was cited as a main reason for why they could not continue chestfeeding. As during the prenatal period, gender dysphoria did not always have to do solely with the individual’s body and what it was doing, but also how the body was being seen and gendered by others.

I was huge, like, it grew like everything came in, so that was dysphoric and I didn’t know what to do with it. I was producing a ton of milk. I mean I could have nursed her fine you know but I just…I didn’t have anything ready socially, either. I didn’t have any zip-up binders. I had no way to stop the milk from leaking through my chest. I had no appropriate clothes for, male clothes for nursing. I didn’t have any of it so and it was just too much to organize on too little sleep. [Emmett, previous double incision without nipple grafts]

I was like, “He needs to eat!” It’s like these two things warring inside of me. One, I’m transgender, but two, I’m a really big proponent of attachment parenting. I think that it’s awesome. I think that breastfeeding and babywearing and family bed and all that stuff really help. [Adam, began coming out and transitioning before pregnancy, presented during pregnancy as female]

I would sometimes go, OK, I’m supposed to be like baby’s so sweet, and we’re bonding, and I was like, whatever, eat. [Henry, came out to himself and others and transitioned after chestfeeding was finished]

Um, even with the nursing I don’t think I’m going to do extended. Like, I might last a couple months but you know I’m going to want to be back on testosterone and because I present as female during the pregnancy and I can only live my life as being seen as female for so long. [Emmett, planning for an additional future pregnancy]

Privacy during chestfeeding was often important to participants. Two participants mentioned in interviews that the need to chestfeed without anyone witnessing, judging, or gendering the process conflicted with their ability to seek chestfeeding support and help.

I spent a lot of time in the room by myself for probably the first 3 months. Every time I needed to feed her I would just go away and do it because I just didn’t feel like I could do it in front of other people. [Peter, no previous chest masculinization surgery; presented as female to all health care providers except his doula]

So I tried [to chestfeed] a few times but I had this like intense privacy about it. Like I didn’t want anyone to know, I didn’t want anyone to see and it just didn’t work out with bringing her home and the in-laws and everything. [Emmett]

I was feeling really inadequate because I had chest surgery and stuff and so anytime she wouldn’t latch I would get really upset. I was really heavily dependent on him [husband] and for a while I wouldn’t try to get her to latch if he wasn’t there…I decided not to [get help from a care provider] because it was so hard that I just couldn’t… I knew that I couldn’t deal if someone ended up being, “Well, of course, she’s not latching.” I pictured the worst and I couldn’t do it. You just, you are putting your trust, and you just never know. [Kai, previous keyhole chest masculinization surgery; did not plan to chestfeed; learned how to use a supplementer from reading a blog post]

Out of the 16 participants who initiated chestfeeding, nine reported that they did not experience any gender dysphoria as a result of the act of chestfeeding. Four participants mentioned in interviews that they were comfortable chestfeeding their babies in public spaces. Again, the participants cited the utility of lactation to fulfill the needs of their babies. One participant expressed the belief that chestfeeding in public is an important, positive act from a social and political perspective.

In the beginning, I was really worried about how that was going to work, like if I was going to be OK with it [chestfeeding]. I have a tendency to cover up in multiple layers and don’t really reveal myself in public. I was really worried about feeding him in public and around friends and family but I think what was kind of interesting to me was that once she was born it just kind of became this thing I needed to do. [Julian, no previous chest masculinization surgery]

I actually really liked it [chestfeeding]. Again, I saw it as a physical process that my body was able to do. Like with the pregnancy, I didn’t gender it. I was like OK, my body can do this, and it actually feels good and I know that my baby is getting something out of it. It was really relaxing. It was nice. [Gabby, came out and transitioned after chestfeeding was finished]

I was happy to be able to use ’em you know. I always said it sure would be nice to take ’em off and put ’em on a shelf you know. For me it was great to be able to use ’em for their intended purpose. [Nick, came out and transitioned after weaning]

Sometimes I kind of covered up. I really liked to wear a button down shirt with an undershirt underneath it and then I could button my shirt down a certain amount and then pull up my undershirt a little bit like an A-shirt…I do think that it’s something [chestfeeding in public] that people should be doing, or that there should be more public space for feeding kids generally and so I was willing to put myself out there a little bit because it’s something that I feel strongly about on a social and political level. So, yeah, sometimes I’d get looks but I didn’t really get many comments and people didn’t really make gendered comments to me about feeding my kids. Yeah, because I think at that point I wasn’t really passing at all. [Vince, no previous chest masculinization surgery]

Some participants had to cope with physical challenges in the postpartum period that were specifically related to medical aspects of their transition. Two participants who had previous chest masculinization surgery experienced engorgement and early signs of mastitis for which they and their health care providers were ill-prepared. One participant who had previous double incision surgery with nipple grafts described his struggle to latch his baby.

It’s a good thing I had a doula because my OB/GYN didn’t say anything about like you need to work—I assumed there wouldn’t be anything to dry up. But actually I did and I almost got mastitis. I started to get sick. I had a lot of pain and I started to get red, streaky hot spots. That’s the mastitis thing. And I started to get a fever. And they, my friend who’s a doula just came and brought all these cabbages and did all this stuff. And I had to get a night doula to get Benadryl some nights too because the, all the other stuff just wasn’t working fast enough. [Felix, previous double incision with nipple grafts; chose before the birth to bottlefeed]

With the follow-up check with the pediatrician, I had developed this mass you know on the left side that was really scary. It was rock hard it was like about this big and I didn’t know what it meant or if it was safe, or what the deal was. So we asked at the doctor and the midwives and we had them check it at the clinic when we took the baby when she was like a week old and they said like well then they recommended not nursing. [Emmett]

I only got him to latch on once for like 10 s and it was glorious. I was like, oh my God, at least I got to feel that at least—I got to experience that little bit and I’ll never forget it but beyond that he never would…Because it was my midwife [who was providing assistance with latching], because it was her, and we had such a good relationship, it was great. I mean it was, you know, she so obviously had my best interests at heart and she cared for me personally and for my baby and for our family in general so I felt very open; I wasn’t scared or nervous. I would have been probably with anybody else. [Cory, previous double incision with nipple grafts]

Two study participants who had planned not to chestfeed due to lack of chest tissue, as well as anticipated gender dysphoria, reversed these decisions after their babies were born.

After she was born, you know she had kind of like crawled up on my chest and I felt the instinct to nurse her which I had really like I mean I had planned so much against it that I had, they had the medication on hand to make the milk dry up…[Emmett, previous chest masculinization surgery]

She was born on a Sunday, yeah, I would say it was a Wednesday when they [mammary glands] got all like puffy. Yeah and I hadn’t tried to see if anything—like it hadn’t occurred to me before that if I could squeeze them and see if…It was my midwife who came over and told me to do that…We were using a bottle and that’s kind of, yeah. It wasn’t until I realized I was producing milk that I was like, oh my God, I want to feed my baby this way and I really want to make this work…[Kai, previous liposuction and keyhole surgery]

Participants commented about how lactation-related care could be made transgender-competent. Some participants reported that they felt respected as transgender patients by individual perinatal providers and that they did not need to educate their providers in terms of language or identity. Two study participants described being touched on their chest without consent, an experience that caused distress during an already challenging time. Participants suggested a need for health care providers to communicate respect for different feeding choices other than chestfeeding, and that providers should neither assume a desire to chestfeed nor push for it.

At one point I was trying to get her to latch and she had just latched when the nurse came in and she was like “Oh, oh, I’ll leave you guys,” and then she came back a bit later and was like, “I’ll talk you through breastfeeding and help you,” and she used “dad” the whole time and “chest.” And I never asked her to do that. She just she was like, “Yeah, you wanna have her on dad’s chest,” and that was the language she used so I thought that was pretty cool. [Kai, previous liposuction and keyhole surgery]

About the nurses, one challenging thing after birth was there’s this huge push to like get the baby latched on and work on feeding and that was challenging because people didn’t really ask if they could grab my breast. You know, they would just grab and manipulate, and um that was pretty alarming. [Julian, no previous chest masculinization surgery, presented as female to care providers]

I think there’s a real push towards breast is best, which I think is great for a lot of reasons, but I’d also want to see health workers have in mind that there are a variety of reasons why people might not nurse and that a lot of those reasons are valid and that gender issues might be a valid reason not to nurse. [Lee]

Study participants discussed their use of donor milk. Seven participants used donor milk or said they were considering using it, and one donated to babies in need. One of these participants mentioned that he received donor milk through a care provider, while another cited a lack of information from health care providers regarding donor milk. One participant recounted that his care providers did not provide adequate information about bottle-feeding. Only one study participant used an at-chest supplementer to feed his baby. Two participants described how they bonded with their babies through close bottle-feeding.

I guess I do have a little bit of a regret. I did have an SNS [supplementer] system that I had in the cabinet for months before my son was born and, in the end, I didn’t even try it. I don’t know why. I think I had put so much effort into trying to produce a little bit of my own milk, and going so far as having blisters from pumping and all that. I had that little weird gadget as I saw it, and I just couldn’t see, you know, if I’m not producing any of my own milk, I just didn’t see using that. [Cory, previous double incision with nipple grafts]

I’ve felt like I could get more information [about infant feeding and using a supplementer] from other trans guys, so with them [health care providers] I focus on the pregnancy and the birth and get me through this. I just don’t want to add anything else to it…I was interested in the supplemental system, like at-the-chest sort of thing, but there was way too much going on to really try. I was really like, I don’t, just put the bottle in his mouth…I was trying to learn a lot of things, cloth diapering, it’s a lot. [Oren, previous double incision chest masculinization surgery]

I didn’t know anything about people having donor milk through milk shares locally or anything like that. I would have been scrambling. That’s another thing that trans people should be made aware of about during their prenatal care, that that’s available and how to educate about that. I don’t know if doctors would talk about that. If it’s too much of a liability to talk about it because of the aspect where you are responsible for screening people and making decisions where you think milk could be contaminated or not. [Adam, speculating about what he would have done if he did not have a full milk supply]

When she has a regular feed like from a bottle she takes about six ounces but when she takes it from me, uh the amount that she takes from me, ’cause you know I use a bottle with the tube [homemade supplementer], she usually only takes about four. So my guess is in each feed she’s getting about one to two ounces. [Kai, previous liposuction and keyhole surgery]

We get the closeness with the bottles. Even now when baby has her feedings, she’s so particular about it. You know, we have to go into the bed, or the quiet place and she has to cuddle, and you know in fact we often call it nursing anyway because it’s not my chest but it’s still the same action from me. [Emmett]

Along with the physical need for donor milk due to chest masculinization surgery or gender dysphoria, there were social aspects to milk sharing as well. Just as one participant made a decision about chestfeeding in the context of his multiracial family and broader community, another participant discussed milk sharing from his perspective as a black, transgender person. The participant reported deciding to come out as gay but not as transgender to his milk donors, and he wryly noted his experience as a black, gay parent receiving milk from many white donors.

I went on to Human Milk 4 Human Babies and I said we were two gay guys—I didn’t mention I was trans—and women were throwing themselves at us, like they got progressive points for giving the gay dudes breast milk…[Partner’s] mom bought us a chest freezer for Christmas ’cause she was tired of us putting milk in hers. He’s loving it, he’s fat, and I’m happy. Everybody’s getting sick, and he’s had a cold, but that’s pretty much it. I always say he’s got the antibodies of like 18 different white women so he’s good to go. [Oren, Black]

#### Later stage

Three participants chestfed for under 6 months, 11 participants chestfed for more than 1 year, and two participants were in the early postpartum period at the time of their interviews and were still chestfeeding. As chestfeeding went on, participants had to make choices about how to balance feeding their babies with gender dysphoria, mental health, and the need to transition.

Two study participants considered binding their chests while they were still chestfeeding but explained that they were unable to do so successfully due to discomfort and a fear of causing mastitis. However, one participant (pseudonym “Adam”) did practice binding and also took testosterone while he was chestfeeding, and he reported these as positive choices. Prior to his pregnancy, Adam had not had chest masculinization surgery and had taken testosterone for under 1 year. He noted that binding and taking testosterone allowed him to present as male and also chestfeed his child into toddlerhood. Both his gender presentation and chestfeeding past infancy were important to this participant. Adam identified the way hormones made him feel as a source of gender dysphoria. He also cited the way others perceived him based on his appearance as a trigger of gender dysphoria.

I didn’t want to force wean him at all. It was just getting to a point where I—I mean it’s such a nebulous feeling, it’s difficult to describe but it’s like you just feel inside what your hormonal dysphoria is, if you have dysphoria around your hormones, and you just know, this is where it’s going to be really bad now, from now on, I’m going to be miserable. [Adam]

In general with transition, I find that when you try to ask people to respect your name and pronouns and who you are, but if you don’t have facial hair and your features are really such that they are going to read you as a woman all the time, it’s really hard for me to get people to take it seriously, and even when they were acting respectful toward me it was like they were doing it to please me but they didn’t really see me the way that I am and it just started to bother me more and more. I was trying to keep my chin up and be really optimistic and I think I succeeded with that for a long time but it was just like you know what, enough is enough. [Adam]

Adam began binding with caution when his baby was approximately 10 months old. He would bind for short periods of time (i.e., an hour or less) and pay close attention to sensations of fullness or discomfort in his chest. He gradually increased the duration of binding sessions and did not experience any problems such as mastitis.

The binding that I use, it’s made of really flexible, elastic material so it shifts around. It’s got this zipper…It doesn’t have Velcro, it doesn’t have much compression. It’s really good for nursing. At first I didn’t like it ’cause it was short and it reminded me of a bra and I thought I’d rather have something like an undershirt but for nursing it’s perfect because I can just unzip it discreetly in a public place and feed him and then zip it up. People don’t necessarily realize that he’s eating…He knows how to unzip my binder. He’s just sort of like, “It’s time for me to eat.” [Adam]

To re-start testosterone therapy, Adam consulted with his endocrinologist and his child’s paediatrician. Adam reported that his doctors referred to a study of low-level testosterone use in lactating, cisgender women to assess safety [33]. He stated that his doctors recommended re-starting testosterone therapy and watching the child closely for any signs of early puberty such as body hair. Adam did re-start testosterone therapy when his child was approximately 21 months old. He reported that approximately 15 months after he had re-started testosterone at a standard dose for female-to-male therapy, blood tests showed normal testosterone levels in his child. He did not notice a decrease in his milk supply that coincided with re-starting testosterone.

I think that getting top surgery and just starting to really feel just this tangible feeling to how much time I have to wait because it was going to be 6 months after he weaned [before surgeon would advise having chest masculinization surgery] and knowing that he could nurse for a long, long time. That actually really drove me with the testosterone decision because it’s going to be a while before I can do something else to relieve things. But between binding and testosterone, that puts me in a really good place with things. [Adam]

Participants described a wide range of thoughts and feelings about getting chest masculinization surgery after weaning was finished. One participant used the surgery as a reward for reaching his personal chestfeeding goals. Six participants described getting chest masculinization surgery as a simple decision once their mammary tissue was no longer being used to nourish their children.

In my mind it [chest masculinization surgery] was like a reward. I felt like if I could at least chestfeed her for the first year then it meant that I could get top surgery, so it was something that I used as like a bit of a, I guess a goal, or something to work towards. [Peter]

I want it [chest masculinization surgery] to happen as soon as possible. Because I pretty much I have the feeling that the breasts were good only for feeding my babies. Otherwise I don’t need them at all…Here I go often at the nude beach and when I am walking there I often I am just crossing my arms so that people couldn’t see the breasts just like a habit or I don’t know because I don’t care if they look at me on the bottom, I, it doesn’t matter but the breasts somehow I feel like they don’t belong there. [Ron]

They were used for their intended purpose. No, I was happy to get rid of them. [Nick]

I feel glad that we’re not planning, like I’m not planning to carry another kid, so I don’t feel like it’s this either or decision [to have chest masculinization surgery or to chestfeed] for my child. So, which I know, definitely know guys who are making that sort of either or decision for kids that they intend to carry. So I’m really glad I don’t have to do that. [Henry, had chest masculinization surgery after weaning was finished]

Three participants mentioned that they never experienced gender dysphoria while chestfeeding but did experience intense gender dysphoria soon after weaning. For these participants, having mammary tissue and using it to feed did not seem to be problematic. However, when their chests were no longer a source of nourishment and others identified these individuals as female because of their chests, intense social gender dysphoria was triggered. One participant did not feel a need for surgery and opted to bind after weaning.

You know it [breastfeeding] was absolutely the best time of my life, even now… As soon as I was done breastfeeding within about a month my dysphoria came back to the point where I couldn’t handle it…It [choosing chest masculinization surgery] was a tough decision for me because I did have such emotional ties because of my experience with my son and the fact that I did want more kids and I always wanted to breastfeed my kids. But, because I was so large chested I knew that this was something I had to do. Because I already had a full beard, I was passing as male. I couldn’t live with the chest I had and I also couldn’t bind…They [chest] were so large. And through the summer it was just too much. I couldn’t eat with the binder on. It was causing other health problems. So I made the decision I had to make even though my chest dysphoria was never terrible. So I went for surgery. [Alex]

I did not [experience gender dysphoria while chestfeeding]. No, mm mm. I was and still am, I mean I’ve just had top surgery, but my, my chest was not a huge source of body dysphoria for me. It was something that, if we lived in a different world that was more accepting of just different identities and different bodies I’m not sure that I would have gotten top surgery. But since you know it’s this sort of thing where people are going to question my identity as a man because of my chest and I wanted to be able to go to the pool or whatever and take off my shirt. Like I really like to swim and I felt really vulnerable whenever I would go swimming, like everyone is seeing me as a woman when I go swimming. In the suits that I have to wear, even though I wear a t-shirt or whatever, I just felt like, yeah, I just felt like so vulnerable like everyone sees a woman right now so that was where that decision came in to get top surgery and it’s been fantastic. [Andrew]

I was very conflicted about potential top surgery, and in the end I’ve decided not to get it but for other reasons. At first I was like I don’t know if I could ever do that because yeah I fed my baby with these…That concern kind of eventually faded because I nurture him in other ways now. I don’t want to put myself through unnecessary surgery anymore. I’m OK with them being there. I’ve found some comfortable binders, and I’m like you know what, that’s fine. [Gabby]

## Discussion

It is apparent from the findings that health care providers working with transgender clients require a nuanced understanding of gender dysphoria that goes beyond the familiar “trapped in the wrong body” narrative. Media depictions of gender dysphoria have treated it as a condition to be cured by medical transitioning and by conforming to societal expectations of traditional gender roles [31]. This strategy may be best for some transgender individuals, but not for all [31]. ‬‬‬‬‬‬‬‬‬‬‬‬‬‬‬‬Participants in this study did not necessarily equate lactation and chestfeeding with femininity, and for those who did, they sometimes opted to chestfeed while finding ways to cope with gender dysphoria if they experienced it.

The experiences of transmasculine individuals reported here show that chest-related gender dysphoria frequently led to chest masculinization surgery and affected feeding choices. Some individuals cited others’ gendered perceptions of their chests and chestfeeding choices, as well as care providers’ physical touching of their chest without permission, as triggers of gender dysphoria. Dutton and Fennie [19] also found that having prominent mammary tissue was a particularly intense source of gender dysphoria in the transgender men they interviewed. For others in our study, there were ways to cope with the dysphoria and reasons such as infant nutrition that made it worthwhile to chestfeed. In another variation, some transgender individuals in our study reported that the act of chestfeeding seemed to mitigate their gender dysphoria because of its utility.

The issue of gendered language was raised frequently by the participants and it has been addressed by other authors [15, 24]. In addition to preferred pronouns, one should consider using masculine or gender-neutral language such as “chestfeeding,” “parent,” and “parental” instead of “breastfeeding,” “mum,” and “maternity.” In North America, “nursing” could substitute for “breastfeeding.” Providers need to be aware that the act of breastfeeding or chestfeeding is not necessarily perceived as feminine by their transmasculine clients.

From the interviews, we see a distinction between gender dysphoria rooted in the individual’s feelings about their body versus gender dysphoria triggered by social interactions. This distinction has important implications for health care providers. It means that care providers and others are capable of *causing* gender dysphoria in a patient by misgendering them. Conversely, care providers can affirm a patient’s gender identity through appropriate language, respectful touch, and other intentional actions, and thus alleviate distress associated with gender dysphoria.

Care providers should use the pronouns and gendered language that patients have stated they prefer, rather than making assumptions based on a patient’s appearance or behaviour. In other words, in order to avoid triggering gender dysphoria, HCPs must *believe the patient* when gender identity is disclosed to them and they must *demonstrate* respect by using appropriate language. As one participant stated and others reiterated in various ways, gender identity is a “two-way street” that involves declaring one’s gender to others, but also having one’s felt gender reflected back to one by others, including health care providers. Care providers could increase their language awareness and competence by practicing the use of gender-neutral language outside of clinical settings and prior to meeting their first transgender clients. We suggest that the ability to use appropriate language is an important skill to be developed, especially in pregnancy and childbirth settings where the norm is to use feminine pronouns and descriptors.

In the academic and medical literature, it is commonly reported that deepening of the voice and growth of body and facial hair, as a result of testosterone treatment, are irreversible in transmasculine individuals [3, 5, 18]. In contrast, we have found this to be true for some, yet for others these secondary sex changes are fully reversible upon the cessation of testosterone treatment. Health care providers should understand that a transgender individual’s gender presentation can revert unpredictably as a result of ceasing testosterone therapy, and that body changes can be distressing for an individual whose gender expression has previously been more masculine.

Bauer et al. report that transgender persons frequently avoid using emergency department services because of previous negative experiences or fear of negative experiences due to their transgender status [17]. We found a similar phenomenon with study participants’ perceptions of their care providers’ assumptions becoming a barrier to accessing care. Surgeons were viewed as gatekeepers to chest masculinization surgery who would not be accepting of future plans for pregnancy or chestfeeding in a transmasculine client. As a result, participants did not obtain information that they required. Two participants avoided asking for lactation help because they had strong needs for privacy during chestfeeding or because they perceived that care providers observing them would misgender them or negatively judge their past choices regarding chest masculinization surgery.

To mitigate problems, we suggest that care providers give clients cues early on in the provider-patient relationship to demonstrate that they have a flexible understanding of gender and gender roles in infant feeding and parenting. Surgeons should state in a pre-operative consultation that they are aware that some transmasculine individuals choose to become pregnant or chestfeed after chest masculinization surgery, thereby opening an opportunity for discussion. Transmasculine individuals considering the surgery need information about the possibility of mammary tissue regrowth in the case of pregnancy. Lactation consultants could mention that they understand that transmasculine clients experience a range of gender dysphoria over chests and chestfeeding or sometimes no dysphoria at all, and then ask the individual how they can best be supported in their plans. If touching might trigger gender dysphoria in a client, a care provider could employ hands-off techniques to demonstrate positioning for chestfeeding or the use of a supplementer.

We learned from the participants that HCPs must be educated about chest care for transmasculine individuals during pregnancy and postpartum. Chest binding during pregnancy will likely be uncomfortable and ineffective beginning early on in gestation and continuing throughout pregnancy. Transmasculine people who have had chest masculinization surgery need to be aware that mammary tissue may or may not grow during pregnancy. Chestfeeding, if desired, may be possible if there is enough tissue for a comfortable latch. Some transmasculine individuals do produce milk after chest masculinization surgery, although at-chest supplementation and donor milk or formula is likely to be required. They should be given the tools to successfully establish a chestfeeding relationship, especially around the use of at-chest supplementers.

The study identified a need for HCPs to provide anticipatory guidance to transmasculine individuals regarding postpartum chest health and lactation. Transgender patients should be taught to recognize engorgement, plugged ducts, and mastitis, whether or not they intend to chestfeed their babies, regardless of their history of chest masculinization surgery. Some participants lacked information about engorgement and mastitis because of the incorrect assumption that someone who had chest masculinization surgery would not experience lactogenesis II following birth.

HCPs should be knowledgeable about potential problems that can be caused by constriction of mammary tissue (e.g., constriction from bras or baby carriers can lead to pugged ducts or mastitis) because transmasculine patients might have questions about how to safely chest bind during lactation. In order to cope with gender dysphoria, transmasculine individuals who are chestfeeding might begin binding with caution for short periods after their milk supply has stabilized. Based on the experience of one study participant, others might wish to take testosterone while chestfeeding under the guidance of a physician who can monitor the infant for possible signs of exposure.

The study finds strength in its roots in the transgender community. It benefits from the leadership of a transgender man who birthed and chestfed two children and a research team that includes clinicians who work with transgender and queer clients and patients. The inclusion of participants who transitioned before having children as well as those who transitioned afterwards provides a rich understanding of the many ways that transgender individuals experience pregnancy, birth, and infant feeding.

The key limitation for this study is about transferability. We do not know whether the convenience sample we recruited reflects the general population of transmasculine individuals experiencing pregnancies. All of the study participants were university educated, and the majority were financially secure and in a committed relationship.

Further studies on this topic should attempt to include more low-income individuals and those with less formal education, and they should also aim to better understand geographical considerations. Future research could try to determine the effect of different types of chest masculinization surgery on lactation. As a possible trend to investigate, we did note that the two participants with previous chest masculinization surgery who had significant mammary tissue re-growth and some amount of lactation following birth both had surgeries that preserved the nipple stalks instead of surgeries involving nipple grafts. The experiences and needs of transmasculine individuals who had unplanned pregnancies could be an important topic for future research.

## Conclusions

The information gathered in this study sheds light on a wide range of transmasculine individuals’ experiences with lactation and infant feeding. Study participants shared stories that were personal and nuanced. There is no single experience that is *the* transgender experience of infant feeding. It is apparent that transmasculine individuals who give birth sometimes choose to chestfeed, even when they have had previous chest surgery, while others do not chestfeed their babies for physical and mental health reasons. Care providers should communicate an understanding of gender dysphoria and transgender identities in order to build patient trust and provide transgender-competent care. Further, HCPs need to be knowledgeable about lactation and chest care during binding and following chest masculinization surgery, regardless of the chosen feeding method, through all stages: before pregnancy, pregnancy, and post birth.

## Ethics and consent to participate

This study was approved by the Health Sciences and Science Research Ethics Board at the University of Ottawa, Ontario, Canada. The approval is based on the *Tri-Council Policy Statement: Ethical Conduct for Research Involving Humans* (2nd edition). Participants provided informed consent in writing or verbally on a digital recording after having opportunities to ask questions about the research.

## Consent to publish

The original transcribed interviews (one each for 18 participants and two each for four participants who completed follow-up interviews) were formatted into separate quotes with extraneous information removed. We kept all quotes directly related to the research question and removed any details that might identify any participant. Names were replaced with pseudonyms approved by the participants. When participant quotes include discussions of race, we identify the racial background of the participant using terms they approved. A set of quotes was sent to each participant for final approval, and permission to publish was obtained in writing.

## Availability of data and materials

Additional file 1: Demographics and question guide. This file contains the questionnaire used to collect demographics information and the semi-structured question guide used as a basis for interviews.

Data from the study other than the quotes already included will not be shared in order to protect participants’ anonymity.

## Additional file

Additional file 1: Demographics and question guide. This file contains the questionnaire used to collect demographics information and the semi-structured question guide used as a basis for interviews. (PDF 66 kb)

## Abbreviations

HCP: health care professional
