# Supplementary material for: Transmasculine individuals’ experiences with lactation, chestfeeding, and gender identity: a qualitative study
Source: BMC Pregnancy Childbirth. 2016 May 16;16:106. doi: 10.1186/s12884-016-0907-y (PMC4867534; doi:10.1186/s12884-016-0907-y)
Supplement: Additional file 1: — Demographics and question guide. This file contains the questionnaire used to collect demographics information and the semi-structured question guide used as a basis for interviews. (PDF 66 kb) [file 12884_2016_907_MOESM1_ESM.pdf]

Do you have any questions about the study?

Do you consent to participation in this study as outlined in the information sheet and consent form?

Demographics and personal history

1. In which country do you currently reside?
   ____

2. What is your age? ________________________

3. What is your relationship status?living with a partner............1 currently not living with a partner................2

4. What level of education have you completed?
   ____

5. What is your family income?$40,000 or less........1 Over $40,000......2

6. What was your first spoken language?
   ____

7. What is your ethnic and cultural background?
   ____

8. How many children do you have?
   ____

9. How many pregnancies have you had?
   ____

10. How many miscarriages have you had?

____

11. Are you pregnant now? YES........1 NO......2

12. In what week of your pregnancy did you give birth? First pregnancy: _______________ Second pregnancy: _________________ Third pregnancy: ______________

13. What was your baby's weight at birth? First baby: ______________________(grams or lbs.) Second baby: __________________ Third baby: ______________

14. Have you experienced postpartum depression?If yes, was it officially diagnosed? YES........1 NO......2

15. Did you take testosterone? YES........1 NO......2If yes, for what period of time and at what dosages?______________________________

16. How long after discontinuing testosterone therapy was it before your cycles returned? ___________________ (please specify weeks/months/years)

17. At the time when you conceived, how long had it been since your last dose of testosterone? ___________________ (please specify weeks/months/years)

18. Did you require fertility assistance to conceive? YES........1 NO......2

19. Were you asked to take any medication during pregnancy specifically due to previous testosterone use or any other aspect of being transgender? YES........1 NO......2

Semi-Structured Interview Guide

Pregnancy and birth

These questions will be a base – other questions and further prompts may be asked depending on what the participants discuss.

How do you self-identify in terms of gender and what language/pronouns do you prefer to use for yourself? (PROMPTS: what does your child call you? How do you refer to your role in the birth? How do you refer to your mammary tissue?)

What was your transition like? (PROMPTS: how long ago? top surgery? hormones? what was it like to access care? consideration of future reproduction at the time?)

Tell me what it was like for you to decide to get pregnant. (PROMPTS: adoption considered? worried about potential gender dysphoria? timing? discussions with health care providers?)

How did you feel about being pregnant? (PROMPTS: thoughts about gender dysphoria? changes to chest tissue? interactions with health care providers, family, friends, colleagues, general public? Did you have any fears about the upcoming birth?)

What was your birth experience like? (*if more than one child, must specify* - how was your first birth different from your second? PROMPTS: choice of birth location? strategies relating to gender identity such as helpful images/symbols? preferred labouring position? medical interventions? was privacy important to you? methods of pain control? interactions with health care providers? what would you change if you could?)

What do you think health care providers need to do in order to care effectively for transmasculine individuals experiencing pregnancy, birth and breast/chestfeeding? (PROMPTS: did you need to teach anything to your care provider? how do you think being trans

affected the care you received?)

How in charge of your own health care during conception/pregnancy/birthing/feeding did you feel? (PROMPTS: did you feel empowered by components of your health care through these processes and how? did you feel safe?)

Breast/chestfeeding:

How did you feed your baby? (PROMPT: How did you make this decision?)

If the participant attempted to or did breast/chestfeed

What was it like to breast/chestfeed your baby? (PROMPTS: latching? positions tried? hands-on help from a care provider? gender dysphoria?)

How much milk did you produce? (PROMPTS: Engorgement? Used any gadgets such as nipple shield or supplementer? Did you pump or take Domperidone? Did you use supplementation [if it was needed, what was used...if someone used donor milk, what was their experience accessing it])

How long did you breast/chestfeed? (PROMPTS: how did weaning happen? did your partner breast/chestfeed the baby? did you have good support from friends/family? did you chestfeed in public?)

OR (*if the participant did not choose to breast/chestfeed):*

What went into your decision to not breast/chestfeed?(PROMPTS: Had you considered it? Who did you discuss your decision with? Was gender dysphoria a factor? Was your history of top surgery a factor? Do you have friends or family who breastfed? Was using testosterone a factor?)

Would you breast/chestfeed a future baby? What would be your reasons?
